# Necrotic ulcers in a HIV-positive man

**DOI:** 10.1016/j.jdcr.2023.11.038

**Published:** 2024-02-24

**Authors:** Nina Lucia Tamashunas, Sarah J. Williamson, Evan Wypasek, Katherine DiSano, Stephen Somach, Amy J. Ray, David R. Crowe

**Affiliations:** a Department of Dermatology, Boston University, Boston, Massachusetts; b Department of Dermatology, MetroHealth Medical Center, Cleveland, Ohio; c Department of Internal Medicine, MetroHealth Medical Center, Cleveland, Ohio; d Department of Infectious Disease, MetroHealth Medical Center, Cleveland, Ohio

**Keywords:** brincidofovir, disseminated, ecthyma, HIV, Monkeypox, mpox, necrosis, tecovirimat, ulcer, vaccinia

## Presentation

**Fig 1:**
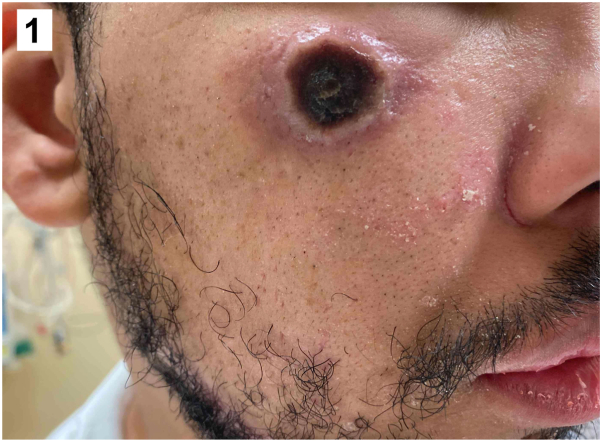
▪

**Fig 2:**
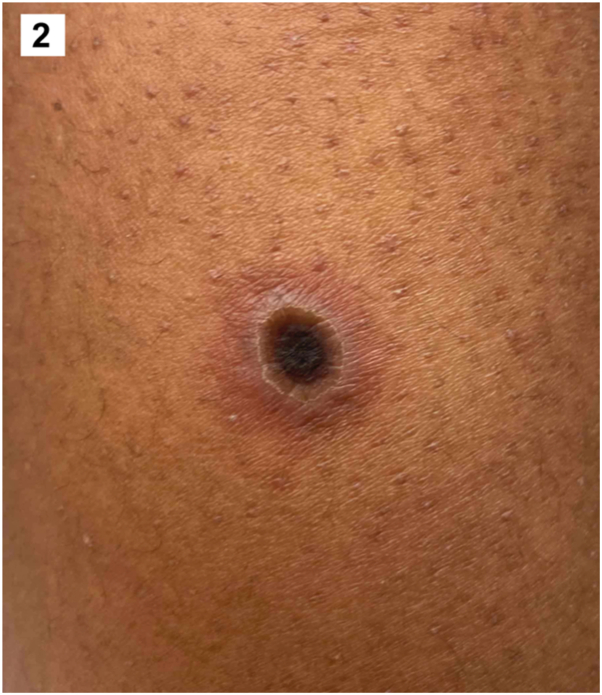
▪

**Fig 3:**
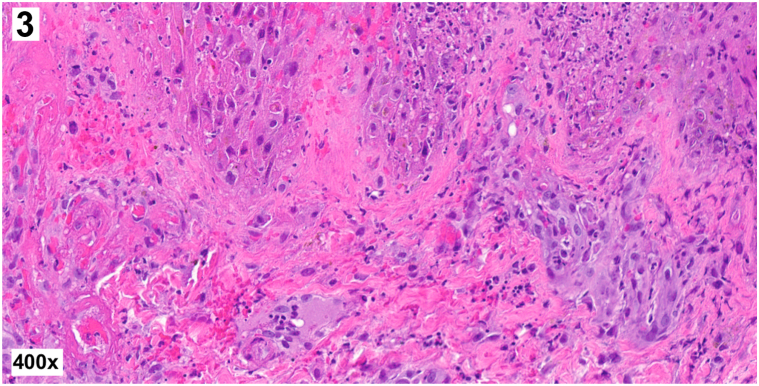
▪

A 30-year-old man with untreated HIV and CD4 count of 59 cells/mm^3^ presented with a 2-week history of fatigue, body aches, fever, and progressive cutaneous ulcers. Physical examination revealed diffuse round necrotic ulcers with pustular borders (Figs 1 and 2), perianal ulcers, and an umbilicated papule on the left buttock. A punch biopsy of a necrotic ulcer on the left arm demonstrated complete epidermal necrosis with ballooning degeneration of keratinocytes, multinucleated keratinocytes, intracytoplasmic eosinophilic inclusion bodies, and foci of neutrophilic vasculitis within the underlying dermis without evidence of bacteria (Fig 3).

**Question 1: What is the most likely diagnosis based on the clinical presentation and histopathology?**

A. Ecthyma gangrenosum
B. Lues maligna
C. Disseminated aspergillosis
D. Mpox
E. Disseminated gonococcal infection

**Answers:**

A. Ecthyma gangrenosum – Incorrect. Given the clinical presentation with round necrotic ulcers, ecthyma gangrenosum was initially high on the differential diagnosis; however, no bacteria were visualized on his biopsy specimen. On histopathology, ecthyma gangrenosum typically demonstrates a blue haze of bacterial bacilli surrounding dermal vessels with associated vessel necrosis.
B. Lues maligna – Incorrect. Lues maligna, also known as malignant syphilis, is a severe variant of secondary syphilis that also can present with a prodrome of fever, followed by ulcerated papules and nodules. However, umbilicated papules are not characteristic. The histopathology of secondary syphilis is variable but can demonstrate lichenoid and perivascular lymphohistiocytic inflammation with elongated slender rete ridges, endothelial swelling, and plasma cells.
C. Disseminated aspergillosis – Incorrect. A disseminated fungal infection such as aspergillosis was initially considered given the patient’s severely immunocompromised status, but histopathology would show narrow septate hyphae with bubbly cytoplasm and 45° angle branching.
D. Mpox – Correct. The diagnosis of mpox was made based on a punch biopsy specimen, which showed typical histopathologic findings of mpox,^1^ and confirmatory polymerase chain reaction (PCR) swab.
E. Disseminated gonococcal infection – Incorrect. Disseminated infection with *Neisseria gonorrhoeae* typically is also associated with arthritis, tenosynovitis, dysuria, and purulent urethral discharge. Histopathology demonstrates a neutrophilic vasculitis but would not show the ballooning degeneration and multinucleated keratinocytes seen in our patient’s biopsy specimen.

**Question 2: Which of the following is the most appropriate treatment in this clinical setting?**

A. Acyclovir
B. Tecovirimat
C. Remdesivir
D. Oseltamivir
E. Ganciclovir

**Answers:**

A. Acyclovir – Incorrect. Acyclovir is a guanosine analog that is approved by the US Food and Drug Administration (FDA) for treatment of herpes simplex virus and varicella-zoster virus infections. Acyclovir is not used for mpox.
B. Tecovirimat – Correct. There are no FDA-approved medications for mpox. The antiviral medications tecovirimat, brincidofovir, and cidofovir as well as intravenous vaccinia immune globulin have been used.^2^ Tecovirimat inhibits proteins involved in formation of the enveloped virion needed for viral dissemination. Tecovirimat has been shown to be highly effective against mpox^2^ and is the preferred therapy in eligible patients with or at risk of developing severe disease.^3^
C. Remdesivir – Incorrect. Remdesivir is an adenosine analog that inhibits SARS-CoV-2 viral replication. It is FDA-approved for treatment of COVID-19 in hospitalized patients or in nonhospitalized patients with high risk of progression to severe disease. Remdesivir is not a recommended treatment for mpox.
D. Oseltamivir phosphate – Incorrect. Oseltamivir phosphate is an FDA-approved antiviral medication used to treat influenza types A and B. It is not a recommended therapy for mpox infection.
E. Ritonavir-boosted nirmatrelvir – Incorrect. Nirmatrelvir is a protease inhibitor that is packaged with ritonavir, a cytochrome P-450 3A4 inhibitor. This combination therapy is FDA-approved for treatment of mild-to-moderate COVID-19 in patients at high risk of progression to severe disease and is not recommended for treatment of mpox.

**Question 3: Which of the following statements is true regarding patients with untreated HIV in whom severe mpox may develop?**

A. Highly active antiretroviral therapy (HAART) initiation should be delayed until ulcers have crusted over
B. There is minimal risk of concurrent opportunistic infection
C. Mpox treatment should be deferred until confirmatory PCR results are available
D. There is low risk of immune reconstitution inflammatory syndrome (IRIS) upon initiation of HAART
E. Patients should be screened for other sexually transmitted infections (STIs)

**Answers:**

A. HAART initiation should be delayed until ulcers have crusted over – Incorrect. Regardless of CD4 count, HAART should be initiated as soon as possible.^4^
B. There is minimal risk of concurrent opportunistic infection – Incorrect. Mitjà et al^3^ found that 26% of patients with CD4 counts of <100 cells/mm^3^ diagnosed with mpox had a concurrent opportunistic infection.
C. Mpox treatment should be deferred until confirmatory PCR results are available – Incorrect. In a retrospective study, 27% of patients with CD4 counts of <100 cells/mm^3^ ultimately died of mpox^3^; therefore, treatment should be started as soon as possible in patients at risk of severe infection.^4^
D. There is low risk of IRIS upon initiation of HAART – Incorrect. Patients should be monitored closely for IRIS because it has been observed in 25% of patients with HIV and mpox.^3^
E. Patients should be screened for other STIs – Correct. Patients with mpox should be screened for concomitant STIs, particularly syphilis, gonorrhea, and chlamydia.^3^

## Conflicts of interest

None disclosed.
